# Small Bowel Transit and Altered Gut Microbiota in Patients With Liver Cirrhosis

**DOI:** 10.3389/fphys.2018.00470

**Published:** 2018-05-01

**Authors:** Yang Liu, Ye Jin, Jun Li, Lei Zhao, Zhengtian Li, Jun Xu, Fuya Zhao, Jing Feng, Huinan Chen, Chengyuan Fang, Rojina Shilpakar, Yunwei Wei

**Affiliations:** Department of Oncological and Laparoscopic Surgery, The First Affiliated Hospital of Harbin Medical University, Harbin, China

**Keywords:** small bowel transit, liver cirrhosis, gut microbiota, 16S rRNA gene, Child–Pugh score

## Abstract

Disturbance of the gut microbiota is common in liver cirrhosis (LC) patients, the underlying mechanisms of which are yet to be unfolded. This study aims to explore the relationship between small bowel transit (SBT) and gut microbiota in LC patients. Cross-sectional design was applied with 36 LC patients and 20 healthy controls (HCs). The gut microbiota was characterized by 16S rRNA gene sequencing. The Firmicutes/Bacteroidetes (F/B) ratio and the Microbial Dysbiosis index (MDI) were used to evaluate the severity of microbiota dysbiosis. The scintigraphy method was performed in patients to describe the objective values of SBT. Patients were then subdivided according to the Child–Pugh score (threshold = 5) or SBT value (threshold = 0.6) for microbiota analysis. LC patients were characterized by an altered gut microbiota; F/B ratios and MDI were higher than HC in both Child_5 (14.00 ± 14.69 vs. 2.86 ± 0.99, *p* < 0.01; 0.49 ± 0.80 vs. -0.47 ± 0.69, *p* < 0.01) and Child_5+ (15.81 ± 15.11 vs. 2.86±0.99, *p* < 0.01; 1.11 ± 1.05 vs. -0.47 ± 0.69, *p* < 0.01) sub-groups in patients. Difference in the gut microbiota between Child_ 5 and Child_5+ patients was inappreciable, but the SBT was relatively slower in Child_5+ patients (43 ± 26% vs. 80 ± 15%, *p* < 0.05). Compared with the Child–Pugh score indicators, SBT showed stronger associations with bacterial genera. A clear difference in the gut microbiota was observed between SBT_0.6- and SBT_0.6+ patients [Pr(>*F*) = 0.0068, pMANOVA], with higher F/B ratios and MDI in SBT_0.6- patients (19.71 ± 16.62 vs. 7.33 ± 6.65, *p* < 0.01; 1.02 ± 0.97 vs. 0.20 ± 0.58, *p* < 0.01). Similar results were observed between the SBT_0.6- and SBT_0.6+ sub-groups of patients with normal liver function and a Child–Pugh score of 5. SBT was negatively correlated with both the F/B ratio and MDI (*r* = -0.34, *p* < 0.05; *r* = -0.38, *p* < 0.05). Interestingly, an increased capacity for the inferred pathway “bacterial invasion of epithelial cells” in patients, was highly negatively correlated with SBT (*r* = -0.57, *p* < 0.01). The severity of microbiota dysbiosis in LC patients depends on SBT rather than Child–Pugh score. SBT *per se* might be significantly related to the gut microbiota abnormalities observed in patients with LC.

## Introduction

Cirrhosis is an advanced liver disease resulting from acute or chronic liver injury, including alcohol abuse, obesity and hepatitis virus infection. Disturbances of the gut microbiota, featured by increased abundance of potentially pathogenic bacteria and decreased levels of beneficial bacteria, are common in LC patients (Chen et al., 2011; Qin et al., 2014; Schnabl and Brenner, 2014; Bajaj et al., 2015). Consequently, abnormal gut microbiota may contribute to liver disease progression (De Minicis et al., 2014; Tilg et al., 2016). The intestinal microflora is the main source of portal LPS and represents an important prerequisite for the development of liver fibrosis in chronic liver injuries (Paik et al., 2003; Isayama et al., 2006; Seki et al., 2007). Gut microbiota dysbiosis plays an important role in the development of LC-related complications, including bacterial infections, a hyperdynamic circulatory state and hepatic encephalopathy (Garcia-Tsao and Wiest, 2004; Riordan and Williams, 2010).

Although the mechanisms underlying microbiota alterations in LC are not clear, the liver is known to interact directly with gut through the hepatic portal and bile secretion systems (Cesaro et al., 2011). Liver dysfunction has many effects on the gut, including impaired small bowel motility (Chang et al., 1998), reduced bile flow and altered secretion of IgA and anti-microbial molecules (Lu et al., 2011). All of these effects could contribute to dysbiosis (Zeuzem, 2000). Altered SBT has been described in patients with LC, with approximately 35% of these patients exhibiting delayed small bowel residence times (Kalaitzakis et al., 2009; Gupta et al., 2010). However, there are some previous studies on SBT in such patients yielding contradictory results (Madsen et al., 2000; Sadik et al., 2003), probably owing to various methodologies used and the small numbers of patients.

The significance of delayed SBT in the patients with LC remains to be explained. Thus far, the relationship between SBT and gut microbiota in LC has not been studied. Nevertheless, delayed SBT or altered small bowel motility has been suggested to be related to small intestinal bacterial overgrowth (IBO). Furthermore, acceleration of the SBT using cisapride is reportedly associated with IBO resolution in 80% of cirrhotic patients (Pardo et al., 2000). The gold standard method for diagnosing IBO, a disorder characterized by excessive bacterial growth in the small bowel, involves collecting an aspirate from the jejunum and observing bacterial growth in excess of 10^5^ per millilitre. However, IBO does not entirely represent microbiota disturbance. The human intestine harbours up to 10^14^ microorganisms, approximately 80% of which are uncultivated and novel species (Eckburg et al., 2005). Over the past decade, culture-independent methods have been applied in studies of human gut microbiota.

In this study, we conducted 16S rRNA gene sequencing to characterize the gut microbiota and used the scintigraphy method to describe objective values of SBT. Because both dysbiosis and delayed SBT can occur in cirrhotic patients, SBT is conceivably related to the gut microbiota. According to this hypothesis, the objective of the present study was to explore the relationship between SBT and the gut microbiota in LC patients.

## Materials and Methods

### Study Population

All patients were recruited from an outpatient clinic and initially diagnosed with LC by comprehensively reviewing the results of liver biopsies, imaging and laboratory tests in addition to clinical symptoms, physical signs, medical history, progress notes and associated complications (Procopet and Berzigotti, 2017). The Child–Pugh scoring was used to assess the prognosis of cirrhosis (Pugh et al., 1973). Patients with progression to hepatic carcinoma, uncontrolled ascites or encephalopathy were excluded.

The healthy control (HC) group consisted of healthy volunteers who visited the First Affiliated Hospital of Harbin Medical University for routine physical examination. The healthy volunteers and patients were well matched for age, sex and BMI. Participants taking any medication (including lactulose) affecting gastrointestinal motility were asked to stop the medication at least 3 days before the SBT studies. All the patients denied receiving any antibiotics during previous 3 months. The following exclusion criteria were applied to all participants: (a) presence of malignancy, infections, known GI or renal disease or significant respiratory or cardiac dysfunction; (b) diagnosis of diabetes mellitus, untreated thyroid dysfunction or previous gastrointestinal surgery; (c) history of an autoimmune disease such as multiple sclerosis, rheumatoid arthritis, IBS and IBD.

The study was approved by the ethics committee and the radiation safety committee of the First Affiliated Hospital of Harbin Medical University. Written Informed consents were obtained from all the participants. The study conformed to the ethical guidelines of the 1975 Declaration of Helsinki and was registered in the Chinese Clinical Trial Registry on November 15, 2015 (ChiCTR-OOB-15007409).

### Measurement of SBT

Small bowel transit was measured using the scintigraphy technique, which is the gold standard for measuring gastrointestinal transit (Graff et al., 2000; Maurer, 2016). Patients were asked about food allergies and were instructed to fast overnight or for a minimum of 8 h before the procedure. Then, ^99m^Tc-DTPA in water was administered together with an unlabelled standard solid meal. The activity range was 18.5 MBq (0.5 mCi) to 37 MBq (1.0 mCi). Images were obtained using a 128 × 128 matrix with a low-energy all-purpose collimator for ^99m^Tc on emission computed tomography. The photopeak setting was 20% at 140 keV.

Anterior and posterior images were acquired for 60 s at 0, 1, 2, 3, 4, 5, and 6 h, and terminal ileum filling at 6 h was considered for interpretation. The ^99m^Tc-DTPA-labelled liquified meal activity was marked visually in the reservoir area and terminal ileum. Then, a ROI was manually drawn to encompass all activity at the site. If activity progressed into the caecum or colon, the mass was considered to have transcended through the small bowel. Therefore, a larger ROI accommodating the terminal ileum, caecum and colon was drawn to measure all activity that passed through the small bowel. Correct positioning of the ROI was confirmed, and tracer progression was assessed by the quantification of passage through the bowel. A large, manually drawn ROI including the entire abdomen was used to obtain four average total abdominal radioactive counts between 2 and 5 h after all the liquid had exited the stomach and was distributed throughout gastrointestinal tract. The average total abdominal counts were used to quantify the activity required for the liquid to enter the colon and to fill the terminal ileum at 6 h. Terminal ileum filling at 6 h was calculated using the following formula: SBT = total counts in the terminal ileum + colon/average total abdominal counts at 2–5 h after meal ingestion. SBT is typically considered delayed if little (40%) to no activity is present in the terminal ileum reservoir (Maurer, 2015).

### Sampling, DNA Extraction, and PCR Amplification

Each participant provided a fresh stool sample in the hospital that was immediately delivered to the laboratory in an insulated box. Upon collection, the fecal sample was immediately divided into aliquots that were then frozen on dry ice and stored at -80°C until use. Microbial DNA was extracted from the fecal samples using E.Z.N.A.^®^ stool DNA Kits (Omega Bio-Tek, Norcross, GA, United States) according to the manufacturer’s instructions.

The V3-V4 hypervariable regions of the bacterial 16S rRNA gene were amplified using the primer pairs forward 341-CCTAYGGGRBGCASCAG and reverse 806-GGACTACNNGGGTATCTAAT in a thermocycler PCR system (GeneAmp 9700, ABI, United States). The final DNA concentration and purity were determined using a NanoDrop 2000 UV-vis spectrophotometer (Thermo Scientific, Wilmington, DE, United States), and the DNA quality was assessed by 1% agarose gel electrophoresis. The PCR reactions were conducted using the following programme: 3 min of denaturation at 95°C, 27 cycles of 30 s at 95°C, 30 s of annealing at 55°C and 45 s of elongation at 72°C, and a final extension at 72°C for 10 min. The PCR reactions were performed in triplicate with 20 μL of a mixture containing 4 μL of 5× FastPfu Buffer, 2 μL of 2.5 mM dNTPs, 0.8 μL of each primer (5 μM), 0.4 μL of FastPfu Polymerase and 10 ng of template DNA. The resulting PCR products were extracted from a 2% agarose gel, further purified using the AxyPrep DNA Gel Extraction Kit (Axygen Biosciences, Union City, CA, United States) and then quantified using QuantiFluor^TM^-ST (Promega, United States) according to the manufacturer’s protocol.

### 16S rRNA Gene Sequencing

Sequencing libraries were generated using a TruSeq^®^ DNA PCR-Free Sample Preparation Kit (Illumina, United States) following the manufacturer’s recommendations, and index codes were added. The library quality was assessed using a Qubit@ 2.0 Fluorometer (Thermo Scientific) and an Agilent Bioanalyzer 2100 system. Finally, the library was sequenced on an Illumina HiSeq 2500 platform, and 250-bp paired-end reads were generated.

Raw fastq files were demultiplexed, quality-filtered by Trimmomatic and merged by FLASH with the following criteria: (a) The reads were truncated at any site receiving an average quality score <20 over a 50-bp sliding window; (b) Primers were exactly matched, allowing for 2-nucleotide mismatching, and reads containing ambiguous bases were removed; (c) Sequences with overlaps greater than 10 bp were merged according to their overlapping sequences.

### Bioinformatic and Statistical Analyzes

16S rRNA gene sequencing data were processed using the Quantitative Insights Into Microbial Ecology platform (QIIME; V.1.9.1) (Kuczynski et al., 2012). OTUs were selected using a cut-off of 97% similarity, and the identified taxonomy was then aligned using the Greengenes database (V.13.8). Chimeric sequences were identified and deleted. A rarefaction curve was constructed to prevent methodological artefacts originating from variations in sequencing depth. The α-diversity was measured based on the species richness from the rarefied OTU table. The β-diversity was estimated by computing the Bray_Curtis distance and was visualized through a PCoA. PCA based on the Euclidean distance was performed to visualize the relative distance between groups, and the sample colour was coded by a group set or an SBT value. Permutational multivariate analysis of variance using distance matrices (pMANOVA) was employed to assess the statistical significance between groups (function ‘adonis’ of the vegan package in R) (Flemer et al., 2017). The differential abundance analysis was performed using the Wilcoxon rank-sum test at the phylum, family, and genus levels. Only taxa with average abundance levels >0.2% and sample coverage >10% were included in the differential analysis. Moreover, a prediction model was proposed based on the relative abundance levels of the top 4 genera that were distinctly distributed between the patients and the HC. To evaluate the discriminatory ability of the prediction model, operating characteristic curves (ROC) were constructed, and the AUC was calculated. Two indexes were used for gut microbiota eubiosis and health status evaluation: (a) the Firmicutes/Bacteroidetes (F/B) ratio (Jeffery et al., 2012); (b) the MDI (Gevers et al., 2014), which is defined as the log of the total abundance levels of organisms that are increased in LC divided by the total abundance levels of organisms that are decreased in LC at the family level. The organisms that are increased in LC included *Streptococcaceae, Peptostreptococcaceae, Erysipelotrichaceae, Clostridiaceae*, and *Pasteurellaceae*, and the organisms that are decreased in LC included *Acidaminococcaceae, Porphyromonadaceae, Prevotellaceae*, and *Bacteroidaceae.* Functional composition profiles of the gut metagenomes were predicted from 16S rRNA gene sequences using PICRUSt in the form of level III KEGG database pathways (Langille et al., 2013).

All statistical analyzes were performed using R and SPSS 19.0 software. For comparison of continuous variables, the Mann–Whitney *U* test was used. For correlation analysis, Spearman’s rank test was performed. Multiple hypothesis tests were adjusted using the Benjamini and Hochberg false discovery rate (FDR), and results below an FDR threshold of 0.05 were considered significant. Logistic regression tests were used to regress the F/B ratio and the MDI against PT, ALB and TBIL. All the tests for significance were two-sided, and *p*-values <0.05 were considered significant.

### Study Design

We conducted a cross-sectional microbiota study in LC patients and HC, followed by studies between sub-groups within LC patients. The SBT study was performed only in LC patients. First, we compared patients with a Child–Pugh score of 5 (Child_5) and patients with a Child–Pugh score >5 (Child_5+). Then, we compared patients with SBT value >0.6 (SBT_0.6+) and patients with SBT value <0.6 (SBT_0.6-). Finally, we compared SBT >0.6 (SBT_0.6+) and SBT <0.6 (SBT_0.6) patients within the Child_5 group.

## Results

### Study Population

From December 1, 2015 to December 31, 2016, 44 patients diagnosed with LC met the criteria for our study. Eight patients were excluded because they were reluctant to participate in the SBT study due to its time-consuming nature. Thirty-six patients remained for further study. The etiology of LC included hepatitis B virus infection (*n* = 28), hepatitis C virus infection (*n* = 2), alcoholic LC (*n* = 3), and autoimmune LC (*n* = 3). Twenty healthy volunteers were recruited as controls. The LC patients and HC were matched for BMI, age and gender. The demographic and clinical characteristics of the participants are summarized in **Table 1**. Twenty-five patients had a Child–Pugh score = 5, and eleven patients had a Child–Pugh score >5. TBIL was higher (*p* < 0.01, Wilcoxon rank-sum test) and the ALB was lower (*p* < 0.01, Wilcoxon rank-sum test) in the Child_5+ group compared with the Child_5 group.

**Table 1:** Demographic characteristics of HC and LC (Child_5 and Child_5+).

	LC (*n* = 36)		*p*-Value+	HC (n=20)	*p*-Value^∗^
	Child_5	Child_5+			
	(n=25)	(n=11)			
Age, *y*, median (min–max)	44(34–65)	52(30–57)	0.59	47(32-–60)	0.72
Male/Female, median (min–max)	10/15	7/4	0.28	12/8	0.41
BMI, kg/m^2^, median (min–max)	22.2(18.1–24.2)	21.7(18.2–25.1)	0.68	22.4(18.5–25.4)	0.75
ALT, U/L, median (min–max)	32.1(9.6–68.7)	44.7(12.0–87.1)	<0.05		
AST, U/L, median (min–max)	37.3(24.8–92.7)	55.5(19.7–259.0)	<0.01		
ALB, g/L, median (min–max)	42.1(36.9–51.8)	38.0(26.5–46.3)	<0.01		
PT, *s*, median (min–max)	12.3(10.8–15.4)	13.3(11.3–17.7)	0.11		
TBIL, μmol/L, median (min–max)	18.7(7.2–33.9)	35.0(14.3–52.4)	<0.01		
Child–Pugh score:					
Child class A: 5	25				
Child class A: 6		6			
Child class B: 7–10		5			

*^∗^Comparison between LC (n = 36) and HC (n = 20); + Comparison between Child_5 (n = 25) and Child_5+ (n = 11).*

*Wilcoxon rank-sum test was used to compare age, BMI and clinical indices; Fisher’s exact test was used to compare gender distribution.*

*ALT, alanine transaminase; ALB, albumin; AST, aspartate aminotransferase; BMI, body mass index; PT, prothrombin time; TBIL, total bilirubin.*

### Gut Microbial Dysbiosis in LC Patients With no Obvious Difference Between the Child_5 and Child_5+ Sub-Groups

After filtering, we obtained 1,410,684 reads in the HC group (an average of 70,534 per sample) and 2,435,191 reads in the LC group (an average of 67,644 per sample). The sample size was equalized to 59,413 for each sample using random subtraction. First, the sequencing depths were examined by plotting the rarefaction curve from the Sobs index (Supplementary Figure S1). Most of the samples exhibited plateaus, suggesting that the sequencing depth was adequate. The α-diversity analysis revealed that both the richness index (ACE) and the diversity index (Shannon) were higher in the LC group than in the HC group, but no significant differences were observed (Supplementary Table S1).

**FIGURE 1:**
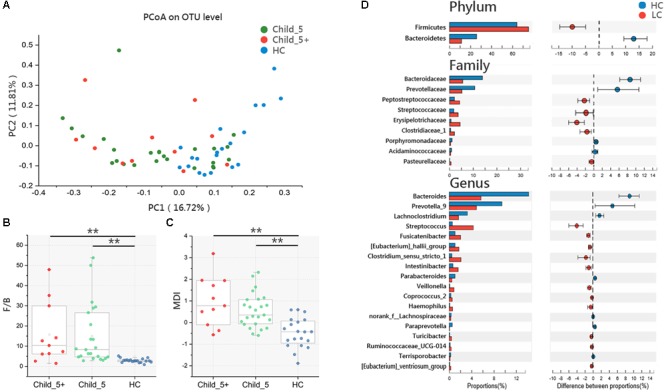
Microbiota study in LC patients and HC. **(A)** PCoA analysis based on Bray_Curtis distance between HC and LC [Pr(>*F*) = 0.0068, pMANOVA], Child_5 and Child_5+ sub-group in LC [Pr(>*F*) = 0.24, pMANOVA]. **(B)** F/B ratio and **(C)** MDI comparison between groups. Boxes represented the 25 to75th percentile of the distribution; the median was shown as a thick line in the middle of the box; whiskers extend to values with 1.5 times the difference between the 25th and 75th percentiles, Wilcoxon rank-sum test, ^∗∗^*p* < 0.01. **(D)** Comparisons of the relative bacterial abundance at phylum, family and genus levels in LC and HC, Wilcoxon rank-sum test, multiple hypothesis tests were adjusted for comparison and only hypothesis tests got *p*_fdr_ < 0.05 were displayed. HC (*n* = 20), LC (*n* = 36, Child_5, *n* = 25; Child_5+, *n* = 11).

Principal coordinate analysis based on the Bray_Curtis distance revealed that the overall microbial composition of the LC group differed from that of the HC group [Pr(>*F*) = 0.02, pMANOVA], but no clear separation was observed between the Child_5 and Child_5+ patients within the LC group [Pr(>*F*) = 0.36, pMANOVA, **Figure 1A**]. The F/B ratio in the HC group was significantly lower compared with those of the Child_5 group (2.86 ± 0.99 vs. 14.00 ± 14.69, *p* < 0.01, Wilcoxon rank-sum test) and the Child_5+ group (2.86 ± 0.99 vs. 15.81 ± 15.11, *p* < 0.01, Wilcoxon rank-sum test, **Figure 1B**). Similarly, the MDI was also lower in the HC group than in the Child_5 group (-0.47 ± 0.69 vs. 0.49 ± 0.80, *p* < 0.01, Wilcoxon rank-sum test) and the Child_5+ group (-0.47 ± 0.69 vs. 1.11 ± 1.05, *p* < 0.01, Wilcoxon rank-sum test, **Figure 1C**). However, the F/B ratios and MDI values were not significantly different between the Child_5 and Child_5+ groups.

We then assessed the relative abundance levels of bacterial taxa in the LC and HC groups. At the phylum level, Bacteroidetes decreased and Firmicutes increased in the LC group (*p*_fdr_ < 0.05, Wilcoxon rank-sum test). Nine taxa at the family level and 18 taxa at the genus level were distinctly distributed between the HC and LC groups (*p*_fdr_ < 0.05, Wilcoxon rank-sum test, **Figure 1D**). In the comparison between the Child_5 and Child_5+ patients, only *Streptococcaceae* at the family level and *Streptococcus* at the genus level showed differences (*p* < 0.05, Wilcoxon rank-sum test without FDR adjustment, Supplementary Table S2). The compositions of bacterial taxa at the phylum, family and genus levels in the three groups are shown in Supplementary Figure S2.

### SBT Was Faster in Child_5 Patients Compared to Child_5+ Patients Within the LC Group

**FIGURE 2:**
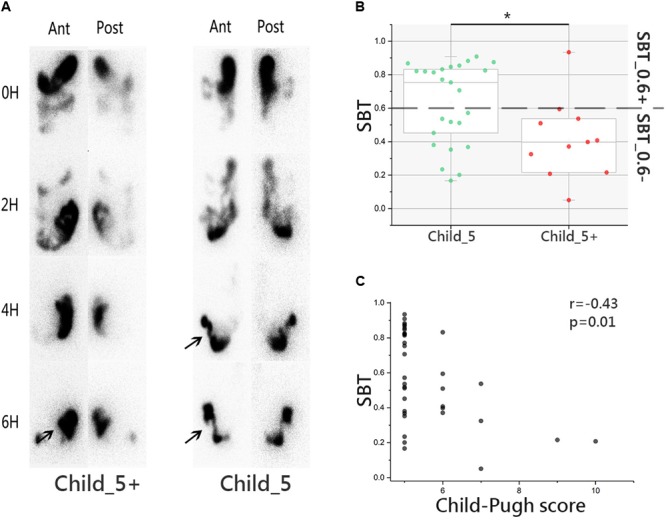
Small bowel transit (SBT) study in LC patients. **(A)** Images of emission computed tomography of paired anterior and posterior images at 0, 2, 4, and 6 h for liquid labeled with ^99m^Tc. Arrow was used to indicate the terminal ileum and cecum-ascending colon. **(B)** SBT value comparison between Child_5 group and Child_5+ group, box plot illustration was provided in **Figure 1**, Wilcoxon rank-sum test, ^∗^*p* < 0.05. **(C)** Correlation analysis between SBT and Child–Pugh score, *r* = –0.43, *p* = 0.01, Spearman’s rank test. LC (*n* = 36, Child_5, *n* = 25; Child_5+, *n* = 11).

The SBT study was conducted in Child_5 and Child_5+ sub-groups within the LC group. Serial images illustrating radiotracer activity in the terminal ileum and caecum of typical patients in the two groups are shown in **Figure 2A**. Over all delayed SBT (<0.4) ratio was 33.3% (12/36). Child_5 showed faster SBT than Child_5+ significantly (43 ± 26% vs. 80 ± 15%, Wilcoxon rank-sum test, *p* < 0.05, **Figure 2B**). 24% of patients in Child_5 group got delayed SBT (6/25), whereas 54.6% patient in Child_5+ group got a delayed SBT (6/11). This different distribution was statistically significant (*p* < 0.05, Fisher’s exact test). Correlation analysis showed Child–Pugh score negatively related to SBT (*r* = -0.43, *p* < 0.05, Spearman’s rank test, **Figure 2C**). But no significant correlations were found between Child–Pugh score and F/B ratio or MDI (Supplementary Figure S3).

### Microbiota Dysbiosis Was More Obvious in Patients With Slow SBT

**FIGURE 3:**
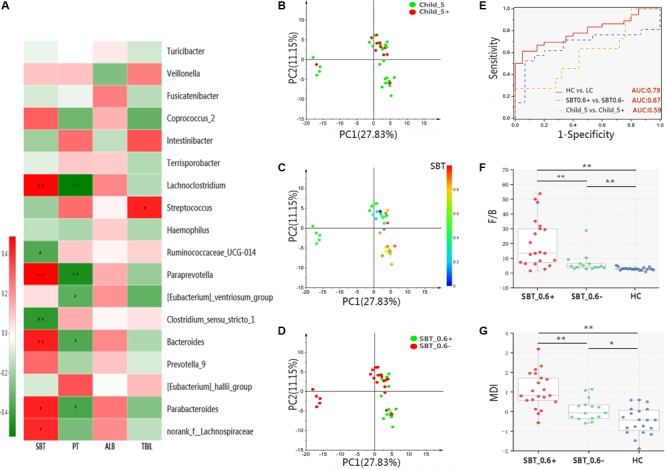
Microbiota study in patients bases on SBT grouping. **(A)** Color-coded Heatmap displaying the relationship between 18 bacterial genera, SBT and Child–Pugh score indicators (PT, ALB, and TBIL). The color scale represents the correlation coefficient. Red: positive correlations; Green: negative correlations, Spearman’s rank test, ^∗^*p* < 0.05, ^∗∗^*p* < 0.01. PCA of samples from LC, color coding base on **(B)** categorical variable of Child–Pugh score [threshold = 5, (Pr(>*F*) = 0.24, pMANOVA)], **(C)** continuous variable of SBT value and **(D)** categorical variable of SBT value [threshold = 0.6, Pr(>*F*) = 0.0068, pMANOVA)]. **(E)** ROC analysis assess the predictive model performance between LC and HC (AUC = 0.79); SBT_0.6– and SBT_0.6+ (AUC = 0.67); Child_5 and Child_5+ (AUC = 0.59). **(F)** F/B ratio and **(G)** MDI comparison between groups, Box plot illustration was provided in **Figure 1**, Wilcoxon rank-sum test, ^∗^*p* < 0.05, ^∗∗^*p* < 0.01. HC (*n* = 20), Child_5 (*n* = 25), Child_5+ (*n* = 11), SBT_0.6– (*n* = 21), SBT_0.6+ (*n* = 15).

A systematic correlation analysis was conducted between 18 discrepant bacterial genera and four parameters, including SBT, PT, ABL, and TBIL, and the results were visualized by Heatmap. Among the four parameters, SBT showed stronger correlations with the bacterial genera than the other three parameters, which are indicators for Child–Pugh scores (**Figure 3A**). Next, a PCA analysis was performed on OTU level in the LC patients subdivided according to Child–Pugh scores or SBT values as described above. Similar to the PCoA results, samples from the Child_5 and Child_5+ groups were not obviously separated [Pr(>*F*) = 0.24, pMANOVA, **Figure 3B**]. Then, samples colours were recoded according to continuous variables of SBT, but the relative locations of the samples remained unchanged. Notably, we found that the SBT values could explain the differences among the samples along the PC2 (**Figure 3C**). Firstly, patients were subdivided based on SBT value with a threshold of 0.4, no obvious separation was observed [Pr(>*F*) = 0.52, pMANOVA, Supplementary Figure S4]. Therefore, sample colours were recoded again based on the categorical variable of SBT at a threshold of 0.6. As expected, the samples could be distinguished between the two groups [Pr(>*F*) = 0.0068, pMANOVA, **Figure 3D**]. In addition, among the bacterial taxa that were distinctly distributed between the LC and HC groups, the relative abundance levels of the two taxa at the phylum level, 7 of the 9 taxa at the family level and 9 of the 18 taxa at the genus level were different between the SBT_0.6- and SBT_0.6+ group (Supplementary Figure S5). A prediction model was proposed based on the relative abundance levels of the top four genera that were distinctly distributed between the LC and HC groups (*Bacteroides, Prevotella_9, Streptococcus*, and *Lachnoclostridium*). The performance of the model in distinguishing LC patients from HCs was assessed using ROC analysis, which achieved an AUC value of 0.79. The discriminatory power of this model was poor for Child_5 and Child_5+ patients, with an AUC value of only 0.59. However, the AUC value was 0.69 for discriminating between SBT_0.6+ from SBT_0.6- patients (**Figure 3E**). Next, we analyzed the F/B ratios and MDI values in LC patients divided into sub-groups according to SBT. The HC group exhibited a significantly lower F/B ratio compared with those of the SBT_0.6+ group (2.86 ± 0.99 vs. 7.33 ± 6.65, *p* < 0.01, Wilcoxon rank-sum test) and the SBT_0.6- group (2.86 ± 0.99 vs. 19.71 ± 16.62, *p* < 0.01, Wilcoxon rank-sum test, **Figure 3F**). The MDI of the HC group was also lower than those of the SBT_0.6+ group (-0.47 ± 0.69 vs. 0.20 ± 0.58, *p* < 0.01, Wilcoxon rank-sum test) and the SBT_0.6- group (-0.47 ± 0.69 vs. 1.02 ± 0.97, *p* < 0.01, Wilcoxon rank-sum test, **Figure 3G**). Interestingly, both the F/B ratio and the MDI of the SBT_0.6+ group were significantly lower compared with those of the SBT_0.6- group (7.33 ± 6.65 vs. 19.71 ± 16.62, *p* < 0.01; 0.20 ± 0.58 vs. 1.02 ± 0.97, *p* < 0.01, Wilcoxon rank-sum test). These significant differences were not observed between Child_5 and Child_5+ patients, indicating that SBT was more closely related to the gut microbiota rather than the Child–Pugh score. Gut microbiota differences between patients can be detected according to SBT variations rather than Child–Pugh scores. The demographic and clinical characteristics of the patients according to SBT sub-groups are shown in Supplementary Table S3.

### Microbiota Differences Can Be Detected in Patients Within the Child_5 Group Based on SBT Sub-Groups

**FIGURE 4:**
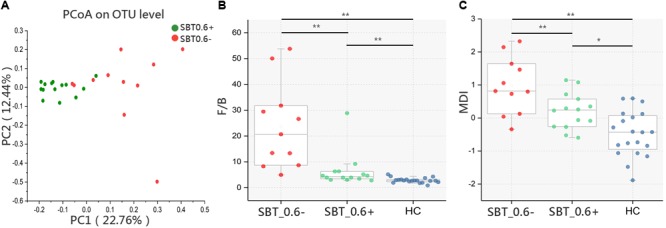
Microbiota study within Child_5 patients base on SBT grouping. **(A)** PCoA analysis based on Bray_Curtis distance between SBT_0.6– and SBT_0.6+ within Child_5 patients [Pr(>*F*) = 0.002, pMANOVA]. **(B)** F/B ratio and **(C)** MDI comparison between groups, Box plot illustration was provided in **Figure 1**, Wilcoxon rank-sum test, ^∗^*p* < 0.05, ^∗∗^*p* < 0.01. HC (*n* = 20), SBT_0.6+ (*n* = 14), SBT_0.6– (*n* = 11).

We showed that microbiota distinction could be achieved within the LC group based on SBT sub-groups rather than on Child–Pugh score sub-groups. However, the PT and TBIL levels between the SBT_0.6+ and SBT_0.6- groups showed significant differences (Supplementary Table S3), which might confound the independent role of SBT in the gut microbiota. To establish an independent correlation between SBT and the microbiota, a study was conducted between sub-groups of Child_5 patients according to SBT. PCoA showed that samples from the SBT_0.6+ and SBT_0.6- groups could be clearly distinguished [Pr(>*F*) = 0.002, pMANOVA, **Figure 4A**]. In addition, among the bacterial taxa that were distinctly distributed between the LC and HC groups, the two taxa at the phylum level, 5 of the 9 taxa at the family level and 10 of the 18 taxa at the genus level were different between fast and slow SBT sub-groups within the Child_5 group (Supplementary Figure S6).

The F/B ratio was significantly lower in the SBT_0.6+ group compared with that in the SBT_0.6- group (7.12 ± 6.85 vs. 21.86 ± 17.50, *p* < 0.01, Wilcoxon rank-sum test, **Figure 4B**). Similar results were also found for the MDI (0.13 ± 055 vs. 0.94 ± 0.86, respectively, *p* < 0.01, Wilcoxon rank-sum test, **Figure 4C**). These differences remained significant after correction for confounding variables, including PT, ALB, and TBIL (Supplementary Table S4). These results further confirmed that gut microbiota differences between patients can be detected according to SBT variations regardless of the Child–Pugh scores. The demographic and clinical characteristics of the patients within the Child_5 group divided according to SBT are shown in Supplementary Table S5.

### Microbial Functions and Correlation Analysis

**FIGURE 5:**
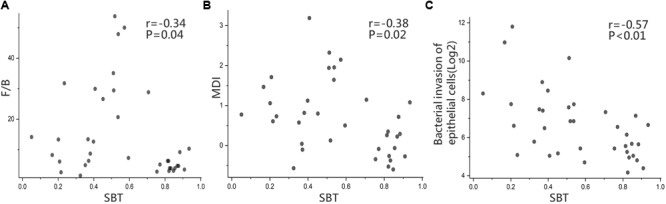
Microbial functions and Correlation analysis. Correlation analysis in LC between **(A)** SBT and F/B ratio (*r* = –0.34, *p* = 0.04), **(B)** SBT and MDI (*r* = –0.38, *p* = 0.02), **(C)** SBT and “bacterial invasion of epithelial cells” pathway (*r* = –0.57, *p* < 0.01), Spearman’s rank test. LC (*n* = 36).

To further evaluate the relationship between SBT and the gut microbiota, we conducted a correlation analysis. The results showed that SBT was negatively correlated with both the F/B ratio (*r* = -0.34, *p* < 0.05, Spearman’s rank test, **Figure 5A**) and the MDI (*r* = -0.38, *p* < 0.05, Spearman’s rank test, **Figure 5B**). Similarly, we also analyzed the relationship between Child–Pugh score indicators (PT, ABL, and TBIL) and both the F/B ratio and the MDI. The Child–Pugh score indicators did not show a significant correlation with neither F/B ratio nor the MDI (Supplementary Figure S7). These results suggested that the severity of gut microbiota dysbiosis depends on SBT rather than the Child–Pugh score and that SBT might contribute to gut microbiota dysbiosis in LC patients.

Biofunctions of the gut microbiota were predicted from 16S rRNA gene sequencing data using PICRUSt in the form of level III KEGG database pathways. One interesting pathway enriched in LC highlighted “bacterial invasion of epithelial cells” although without statistical significance (92 ± 112.35 vs. 85 ± 62, *p* > 0.05, Wilcoxon rank-sum test). SBT showed a significant negative relationship with the relative abundance of “bacterial invasion of epithelial cells” pathway (*r* = -0.57, *p* < 0.01, Spearman’s rank test, **Figure 5C**).

## Discussion

In the current study, we applied 16S rRNA gene sequencing and the scintigraphy method to evaluate the gut microbiota and SBT. LC is characterized by an altered gut microbiota, but no obvious differences were found between samples from Child_ 5 and Child_5+ patients. SBT was relatively slow in Child_5+ patients. The degree of microbial dysbiosis was found to be more obviously associated with SBT rather than the severity of liver function impairment.

Despite the relatively low Child–Pugh scores and the inclusion of only five patients with a level B Child–Pugh score in this study, different microbial compositions between patients and HCs were very evident. At the phylum level, Bacteroidetes was significantly under-represented, whereas Firmicutes was over-represented in the LC patients, which is consistent with the results of a previous study comparing LC patients and healthy subjects by 16S rRNA gene sequencing (Chen et al., 2011). We used two indexes for gut microbiota eubiosis evaluation, the F/B ratio and the MDI, which are considered representative of the health status and indicative of eubiosis of the gastrointestinal tract (Jeffery et al., 2012; Gevers et al., 2014). Both indexes were higher in the LC patients than those in the HC.

A previous study showed that the severity of liver function impairment was correlated with the degree of microbial dysbiosis. Microbiota dysbiosis in compensated (Child–Pugh levels A and B) LC patients is milder than that in decompensated (Child–Pugh level C) LC patients (Qin et al., 2014). IBO is also common in cirrhosis (Casafont Morencos et al., 1996; Chang et al., 1998) and appears to be related to liver disease severity (Pande et al., 2009). In a sub-group analysis in patients, because only five patients had Child–Pugh level B scores, we grouped the patients according to Child–Pugh scores using a threshold of 5. Our results showed no significant differences in the microbiota composition or the microbiota eubiosis index between Child_5 and Child_5+ sub-group, which may be due to the relatively small proportion of patients with high Child–Pugh scores included in this study.

Altered SBT has been described in patients with LC, with approximately 35% of patients showing a delayed small bowel residence time (Kalaitzakis et al., 2009; Gupta et al., 2010; Chander Roland et al., 2013). Our study revealed delayed SBT (<0.4) in 12 of the 36 (33%) patients, the incidence rate was similar to that in a previous study, which were both obviously higher than that in HCs which was at approximately 10% (Kalaitzakis et al., 2009). A sub-group analysis showed that Child_5 patients had better SBT than Child_5+ patients, which is consistent with reports from earlier studies indicating a relationship between the severity of liver failure and the intensity of small bowel motility disturbances (Madrid et al., 1997a,b; Chander Roland et al., 2013). In this study, some of the patients with Child–Pugh score 5 nonetheless still exhibited slow SBT. Thus, indicating the obvious role of some other factors affecting SBT in addition to Child–Pugh score. Mechanisms involved for slow SBT in cirrhosis patients are not yet fully understood. Conceivably, portal hypertension itself could significantly related to abnormal small bowel motility in the LC patients (Gunnarsdottir et al., 2003). Unfortunately, the portal vein pressure was not measured in this study; thus, we could not evaluate the relationship between the portal vein pressure and SBT. Some other studies have also suggested that abnormal small bowel motility is associated with altered functions of the enteric or autonomic nervous systems (Pardo et al., 2000).

Intestinal bacterial overgrowth is an important indicator in evaluating the gut microbiota in LC and is normally diagnosed by quantitative culture of jejunal secretions. Several circumstances in cirrhosis could predispose a patient to IBO, such as alcohol abuse (Bode et al., 1984), malnutrition (Casafont et al., 1997), hypochlorhydria (Shindo et al., 1993), decreased IgA or bile salts in the intestine (Deitch et al., 1990) and disturbances of small bowel motility (Vantrappen et al., 1977; Runkel et al., 1993). Among these mechanisms, prolonged SBT appears to play a major role in the development of IBO. Furthermore, improving small bowel motility with the use of cisapride has been reported to be associated with IBO resolution (Wang et al., 1996; Madrid et al., 2001). However, IBO is not sufficiently comprehensive to serve as an indicator for assessing gut microbiota turbulence. Until now, no study has evaluated the relationship between SBT and the gut microbiota in LC.

In our study, compared to PT, ABL, and TBIL, SBT showed stronger correlations with bacterial taxa. PCA can distinguish patients more effectively according to SBT on the threshold of 0.6 rather than 0.4 (the threshold for delayed or normal SBT classification). Two reasons may explain this phenomenon: First, the sample size is not large enough to get the statistical significance between two sub-groups; second, there were 9 patients with normal SBT value, between 0.4–0.6, but yet have an LC related fecal microbiota profile. The combination of four bacterial genera distinguished patients from HCs with high accuracy. This model can distinguish SBT_0.6+ patients from SBT_0.6- patients with an AUC of 0.67, but it does not perform well in differentiating between Child_5 and Child_5+ patients, suggesting that SBT categories are more reasonable for detecting gut microbiota differences. In addition, the F/B ratio and MDI were significantly lower in the SBT_0.6+ group, which were close to those in the HCs. Notably, the PT and TBIL levels were significantly different between the SBT_0.6+ and SBT_0.6- patients, which could potentially confound the role of SBT in the gut microbiota. A comparison between SBT_0.6+ and SBT_0.6- patients within the Child_5 group, all of whom had normal liver function, revealed differences in microbiota dysbiosis severity. These differences remained significant even after correction for PT, ALB and TBIL. Furthermore, SBT exhibited significantly negative correlations with the F/B ratio and MDI and on the other hand, PT, ABL, and TBIL had no obvious correlations with the F/B ratio or the MDI. These results suggested that in an LC population with an overall relatively mild level of liver function impairment, microbiota dysbiosis severity was not obviously different between patients with different Child–Pugh scores. However, SBT differences were evident between these patients and were closely related to microbiota dysbiosis severity. Therefore, SBT *per se* might be related to the gut microbiota.

Gut microbiota dysbiosis plays an important role in the development of LC-related complications due to bacterial translocation, such as spontaneous bacterial peritonitis, which can further complicate ascites and abdominal and systemic inflammation and lead to potentially fatal complications, such as variceal bleeding and hepatic encephalopathy (Thalheimer et al., 2005). Analysis of the inferred metagenome identified the LC enriched pathway “bacterial invasion of epithelial cells,” indicating that pathogenic bacteria cross epithelial barriers, colonize cells and invade internal tissues, ultimately leading to bacterial translocation (Tuomisto et al., 2014; Tang et al., 2017). Our study showed that the relative abundance of this pathway was negatively correlated with SBT. These results may explain the findings of a previous study that showed delayed SBT accompanied by bacterial translocation in LC patients (Pardo et al., 2000; Sanchez et al., 2005).

The findings regarding the relationship between SBT and the gut microbiota in patients with cirrhosis are novel. However, several limitations must be addressed in future studies. First, this is a relatively small study with limited sample size, we had no opportunity observe these results among patients with higher Child–Pugh scores as these patients did not meet the inclusion criteria for various reasons, including antibiotic, proton pump inhibitor or H2 receptor blocker application, or could not participate in the SBT study due to their physical conditions. Therefore, this study may not be sufficient to generalize to all patients with cirrhosis. Second, we had not conducted SBT study in HC cohort as the participants collected from a group of patients undergoing routine physical examination, were reluctant to partake in SBT study. We had to encounter with similar situation in previous studies. Reference of normal values were taken from other/alternative researches whereas SBT or small bowel motility tests were performed in patient cohort only (Gunnarsdottir et al., 2003; Kalaitzakis et al., 2009). Third, the design of our study was cross-sectional; thus, statistical correlations between SBT and gut microbiota profiles do not necessarily implicate a cause-effect relationship. We will study the gut microbiota in LC patients before and after cisapride therapy in a further study. Fourth, malnutrition, hypochlorhydria and decreased intraluminal immunoglobulin A or bile salts in the intestine, which have been proposed as factors affecting the microbiota in cirrhosis, were not evaluated in the present study and warrant further investigation. Lastly, the gut microbiota analysis was performed on fecal samples, which are not representative of the small bowel microbiota, although we found a relationship between SBT and the fecal microbiota. The contents of the small bowel are not easy to obtain, but we will conduct further studies to analyse the microbiota in aspirate samples from the jejunum.

Despite these limitations, based on our comprehensive investigation of the fecal microbiome and SBT in LC patients, the assumption that slow SBT *per se* might be significantly related to the gut microbiota abnormalities observed in LC patients is intriguing. Although SBT may not be the single most important reason for an altered gut microbiota in cirrhosis, further studies are certainly warranted. Whether SBT has a cause-effect role in the gut microbiota and subsequently whether manipulating SBT can improve abnormal gut microbiota to prevent LC-related complications represent promising targets for future studies.

## Availability of Data and Materials

Raw sequences have been deposited by us into the NCBI Sequence Read Archive (SRP136505), the necessary metadata can be found at https://www.ncbi.nlm.nih.gov/Traces/study/ and searching the respective SRA study accession. Detailed information of every study subject was exhibited in the Supplementary Table S6.

## Author Contributions

YW, YL, and YJ conceived the project. YL, YJ, FZ, JF, HC, and CF performed the experiments. JL, LZ, ZL, and JX contributed to discussions regarding the project. YL, YJ, RS, and YW analyzed the data and wrote and revised the manuscript.

## Conflict of Interest Statement

The authors declare that the research was conducted in the absence of any commercial or financial relationships that could be construed as a potential conflict of interest.

## Abbreviations

AUC: area under the curve
F/B ratio: Firmicutes/Bacteroidetes ratio
HC: health control
IBO: intestinal bacteria overgrowth
LC: liver cirrhosis
MDI: microbial dysbiosis index
OTUs: operational taxonomic units
PCA: principal component analysis
PCoA: principal coordinate analysis
ROC: receiving operational curve
ROI: region of interest
SBT: small bowel transit
