# Supplementary material for: Small Bowel Transit and Altered Gut Microbiota in Patients With Liver Cirrhosis
Source: Front Physiol. 2018 May 1;9:470. doi: 10.3389/fphys.2018.00470 (PMC5946013; doi:10.3389/fphys.2018.00470)
Supplement: Supplementary file 1 [file Table_1.DOCX]

**Table S1**. Pyrosequencing data dummary.

|  | **LC**  **(n=36)** | **HC**  **(n=20)** | **pValue** |
| --- | --- | --- | --- |
| Sequences number | 67644±5791 | 70534±4093 | P<0.05 |
| OTU | 414±112 | 354±34 | P<0.05 |
| ACE | 448.68±133.01 | 429.56±48.89 | P=0.09 |
| Shannon | 3.847±0.36 | 3.76±0.32 | P=0.14 |

The number of OTUs, richness estimates (ACE), and diversity estimates (Shannon index) were calculated after sequences were number-equalized to 59,413, Wilcoxon rank-sum test.
