# Supplementary material for: Small Bowel Transit and Altered Gut Microbiota in Patients With Liver Cirrhosis
Source: Front Physiol. 2018 May 1;9:470. doi: 10.3389/fphys.2018.00470 (PMC5946013; doi:10.3389/fphys.2018.00470)
Supplement: Supplementary file 2 [file Table_2.DOCX]

| **Table S2**. Statistical details of differential taxa abundances at phylum, family and genus levels | | | | | | |
| --- | --- | --- | --- | --- | --- | --- |
|  | **LC**  **(n=36)** | | **pValue^+^** | **HC**  **(n=20)** | **pValue*** | **p_fdr_** |
|  | **Child_5**  **(n=25)** | **Child_5+**  **(n=11)** |  |  |  |  |
| **Phylum** |  |  |  |  |  |  |
| Firmicutes | 78.45±8.76 | 74.61±15.5 | 0.73 | 65.84±9.82 | <0.001 | <0.001 |
| Bacteroidetes | 11.28±8.31 | 12.17±11.92 | 0.95 | 26.36±8.90 | <0.001 | <0.001 |
| **Family** |  |  |  |  |  |  |
| Bacteroidaceae | 6.33±5.09 | 4.31±3.79 | 0.34 | 14.36±4.17 | <0.001 | <0.001 |
| Prevotellaceae | 4.35±4.49 | 7.55±11.16 | 1 | 10.96±9.71 | 0.002 | 0.006 |
| Peptostreptococcaceae | 4.97±4.52 | 3.25±1.81 | 0.86 | 2.21±1.08 | 0.003 | 0.006 |
| Streptococcaceae | 3.37±4.02 | 7.50±7.37 | 0.02 | 0.58±0.53 | <0.001 | <0.001 |
| Erysipelotrichaceae | 2.45±2.20 | 6.10±10.81 | 0.29 | 1.38±1.85 | 0.02 | 0.04 |
| Clostridiaceae_1 | 2.51±5.34 | 1.20±0.79 | 0.95 | 0.49±0.28 | <0.001 | <0.001 |
| Porphyromonadaceae | 0.60±0.59 | 0.31±0.23 | 0.19 | 1.04±0.44 | <0.001 | <0.001 |
| Acidaminococcaceae | 0.33±0.71 | 0.89±2.44 | 0.60 | 0.79±1.26 | 0.049 | 0.049 |
| Pasteurellaceae | 0.31±0.46 | 1.29±3.29 | 0.20 | 0.12±0.33 | <0.001 | <0.001 |
| **Genus** |  |  |  |  |  |  |
| Bacteroides | 6.33±5.09 | 4.31±3.79 | 0.34 | 14.36±4.17 | <0.001 | <0.001 |
| Prevotella_9 | 4.15±4.46 | 6.58±9.50 | 0.97 | 9.51±9.92 | 0.015 | 0.046 |
| Lachnoclostridium | 1.87±2.23 | 1.25±1.11 | 0.26 | 3.27±1.76 | <0.001 | <0.001 |
| Streptococcus | 3.01±3.29 | 7.42±7.40 | 0.02 | 0.58±0.53 | <0.001 | <0.001 |
| Fusicatenibacter | 2.23±1.45 | 1.61±0.93 | 0.16 | 1.04±0.30 | <0.001 | <0.001 |
| [Eubacterium]_hallii_group | 1.69±0.5 | 1.88±1.26 | 0.90 | 1.07±0.43 | <0.001 | 0.001 |
| Clostridium_sensu_stricto_1 | 2.51±5.34 | 1.20±0.79 | 0.95 | 0.49±0.28 | <0.001 | 0.001 |
| Intestinibacter | 1.78±2.02 | 1.18±0.84 | 0.81 | 0.65±0.43 | 0.003 | 0.014 |
| Parabacteroides | 0.6±0.59 | 0.31±0.31 | 0.23 | 1.04±0.44 | <0.001 | <0.001 |
| Veillonella | 0.82±1.51 | 1.02±1.19 | 0.24 | 0.08±0.06 | <0.001 | <0.001 |
| Coprococcus_2 | 0.57±0.38 | 0.42±0.22 | 0.81 | 0.33±0.28 | 0.009 | 0.034 |
| Haemophilus | 0.31±0.46 | 1.29±3.29 | 0.81 | 0.12±0.33 | <0.001 | <0.001 |
| norank_f__Lachnospiraceae | 0.3±0.24 | 0.26±0.30 | 0.51 | 0.36±0.12 | 0.010 | 0.041 |
| Paraprevotella | 0.09±0.1 | 0.04±0.04 | 0.19 | 0.55±0.49 | <0.001 | <0.001 |
| Turicibacter | 0.46±0.8 | 0.15±0.06 | 0.32 | 0.15±0.27 | 0.010 | 0.040 |
| Ruminococcaceae_UCG-014 | 0.35±0.61 | 0.22±0.20 | 0.90 | 0.08±0.20 | 0.009 | 0.034 |
| Terrisporobacter | 0.51±0.82 | 0.25±0.12 | 0.59 | 0.12±0.15 | <0.001 | <0.001 |
| [Eubacterium]_ventriosum_group | 0.19±0.11 | 0.15±0.07 | 0.37 | 0.30±0.17 | 0.007 | 0.030 |

*Comparison between LC (n=36) and HC (n=20); **+** Comparison between Child_5 (n=25) and Child_5+ (n=11).

Wilcoxon rank-sum test, multiple hypothesis tests were adjusted using the Benjamini and Hochberg false discovery rate (FDR).
