# Supplementary material for: Small Bowel Transit and Altered Gut Microbiota in Patients With Liver Cirrhosis
Source: Front Physiol. 2018 May 1;9:470. doi: 10.3389/fphys.2018.00470 (PMC5946013; doi:10.3389/fphys.2018.00470)
Supplement: Supplementary file 3 [file Table_3.DOCX]

**Table S3**. Demographic characteristics of patients from SBT_0.6+ and SBT_0.6-

|  | **SBT_0.6+**  **(n=15)** | **SBT_0.6-**  **(n=21)** | **p**  **Value** |
| --- | --- | --- | --- |
| Age, y, median (min–max) | 43.0(34-65) | 50.0(30-60) | 0.27 |
| Male/Female | 7/8 | 10/11 | 1.00 |
| BMI, kg/m^2^, median (min–max) | 22.4(18.3-24.2) | 21.7(18.2-25.5) | 0.57 |
| ALT, U/L, median (min–max) | 32.3(9.6-68.7) | 35.7(10.6-111.6) | 0.70 |
| AST, U/L, median (min–max) | 37.1(24.9-73.8) | 45.9(19.7-259.0) | 0.20 |
| ALB, g/L, median (min–max) | 42.4(37.5-49.4) | 40.1(26.5-52.8) | 0.06 |
| PT, s, median (min–max) | 11.8(10.8-13.1) | 12.9(11.2-17.7) | p<0.01 |
| TBIL, μmol/L, median (min–max) | 17.7(7.2-38) | 26.6(10.6-52.4) | P<0.05 |

Wilcoxon rank-sum test was used to compare age, BMI and clinical indices; Fisher’s exact test was used to compare gender distribution.

ALT, alanine transaminase; ALB, Albumin; AST, aspartate aminotransferase; BMI, body mass index; PT, Prothrombin time; TBIL, total bilirubin.
