# Supplementary material for: Small Bowel Transit and Altered Gut Microbiota in Patients With Liver Cirrhosis
Source: Front Physiol. 2018 May 1;9:470. doi: 10.3389/fphys.2018.00470 (PMC5946013; doi:10.3389/fphys.2018.00470)
Supplement: Supplementary file 4 [file Table_4.DOCX]

**Table S4**. F/B ratio and MDI comparison between SBT_0.6+ and SBT_0.6- in Child_5 group.

|  | **SBT_0.6+** | **SBT_0.6-** | **p Value** | **Logistic P** |
| --- | --- | --- | --- | --- |
| F/B ratios | 7.12±6.85 | 21.86±17.50 | 0.001 | 0.030 |
| MDI | 0.13±055 | 0.94±0.86 | 0.004 | 0.028 |

Wilcoxon rank-sum test. Logistic regression test was adjusted for covariates including PT, ALB and TBIL.

F/B ratio, Firmicutes/Bacteroidetes ratio; MDI, Microbial Dysbiosis index.
