# Supplementary material for: Small Bowel Transit and Altered Gut Microbiota in Patients With Liver Cirrhosis
Source: Front Physiol. 2018 May 1;9:470. doi: 10.3389/fphys.2018.00470 (PMC5946013; doi:10.3389/fphys.2018.00470)
Supplement: Supplementary file 5 [file Table_5.DOCX]

**Table S5**. Demographic characteristics of patients from SBT_0.6+ and SBT_0.6- in Child_5 group.

|  | **SBT_0.6+**  **(n=14)** | **SBT_0.6-**  **(n=11)** | **p Value** |
| --- | --- | --- | --- |
| Age, y, median (min–max) | 42.5(34-65) | 46.0(36-60) | 0.30 |
| Male/Female | 6/8 | 4/7 | 1.00 |
| BMI, kg/m^2^, median (min–max) | 22.7(18.3-24.2) | 21.4(18.4-25.5) | 0.54 |
| ALT, U/L, median (min–max) | 32.2(9.6-68.7) | 31.4(10.6-67.1) | 0.67 |
| AST, U/L, median (min–max) | 36.9(24.9-73.8) | 40.1(24.8-92.7) | 0.60 |
| ALB, g/L, median (min–max) | 42.2(37.5-49.4) | 42.1(36.9-51.8) | 0.95 |
| PT, s, median (min–max) | 11.8(10.8-13.1) | 12.6(11.2-15.4) | 0.03 |
| TBIL, μmol/L, median (min–max) | 16.4(7.2-26) | 20.6(10.6-33.9) | 0.18 |

Wilcoxon rank-sum test was used to compare age, BMI and clinical indices; Fisher’s exact test was used to compare gender distribution.

ALT, alanine transaminase; ALB, Albumin; AST, aspartate aminotransferase; BMI, body mass index; PT, Prothrombin time; TBIL, total bilirubin.
