# Supplementary material for: Small Bowel Transit and Altered Gut Microbiota in Patients With Liver Cirrhosis
Source: Front Physiol. 2018 May 1;9:470. doi: 10.3389/fphys.2018.00470 (PMC5946013; doi:10.3389/fphys.2018.00470)
Supplement: Supplementary file 7 [file Image_1.PDF]

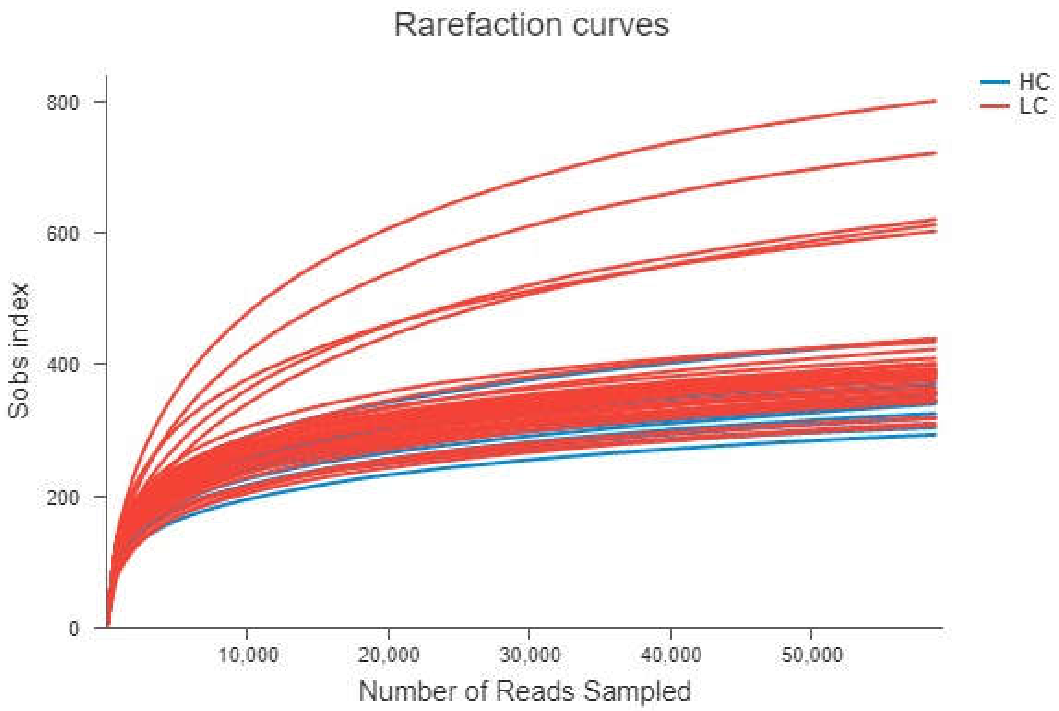

Figure S1. Rarefaction curves of Sobs index on OTU level were used to show all the samples reached plateaus.
