# Supplementary material for: Small Bowel Transit and Altered Gut Microbiota in Patients With Liver Cirrhosis
Source: Front Physiol. 2018 May 1;9:470. doi: 10.3389/fphys.2018.00470 (PMC5946013; doi:10.3389/fphys.2018.00470)
Supplement: Supplementary file 8 [file Image_2.PDF]

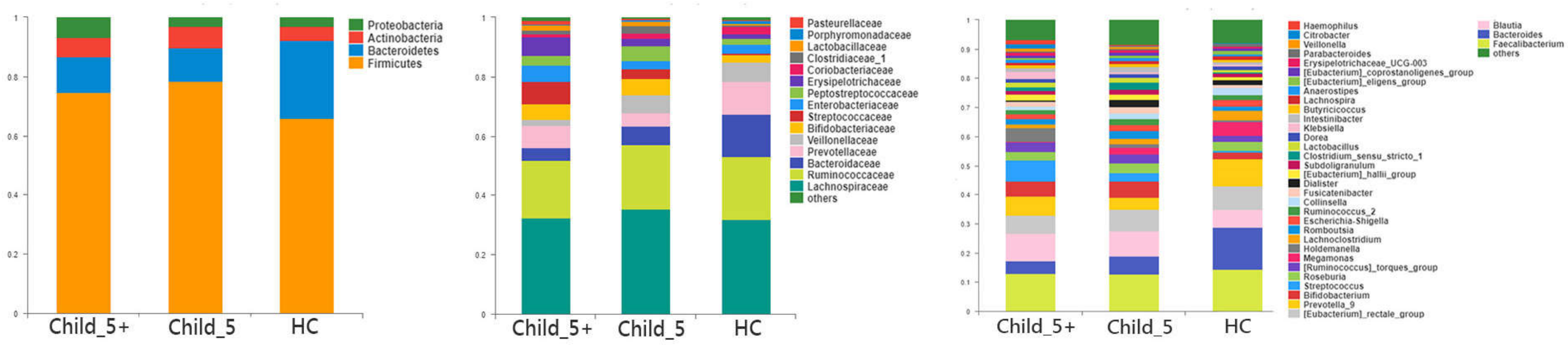

Figure S2. Composition of bacterial taxa on phylum, family and genus level in HC, Child_5 and Child_5+ group. Only relative abundances greater than 1% were included. All OTUs with lower abundances were grouped as “other”.
HC (n=20), Child_5 (n=25), Child_5+ (n=11)
