# Supplementary material for: Small Bowel Transit and Altered Gut Microbiota in Patients With Liver Cirrhosis
Source: Front Physiol. 2018 May 1;9:470. doi: 10.3389/fphys.2018.00470 (PMC5946013; doi:10.3389/fphys.2018.00470)
Supplement: Supplementary file 10 [file Image_4.PDF]

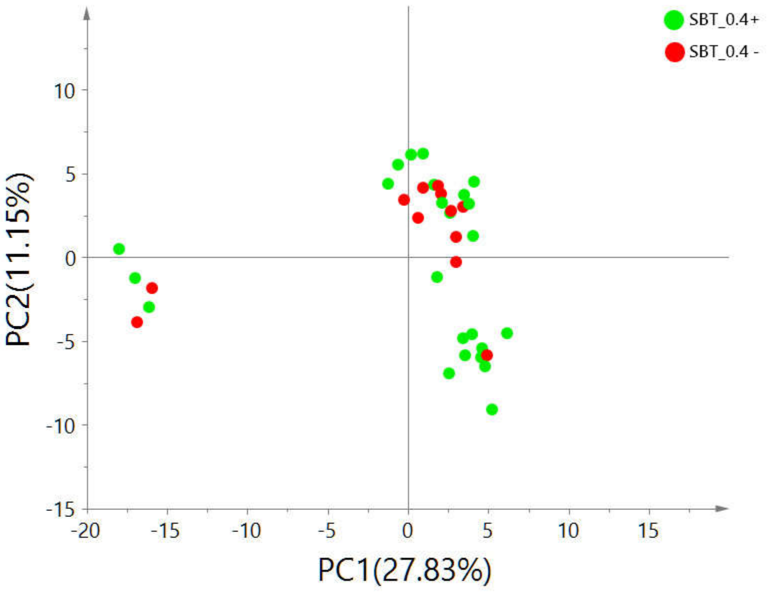

Figure S4. PCA of samples from LC, color coding base on categorical variable of SBT (threshold=0.4, (Pr(>F)=0.52 , pMANOVA)
