# Supplementary material for: Small Bowel Transit and Altered Gut Microbiota in Patients With Liver Cirrhosis
Source: Front Physiol. 2018 May 1;9:470. doi: 10.3389/fphys.2018.00470 (PMC5946013; doi:10.3389/fphys.2018.00470)
Supplement: Supplementary file 11 [file Image_5.PDF]

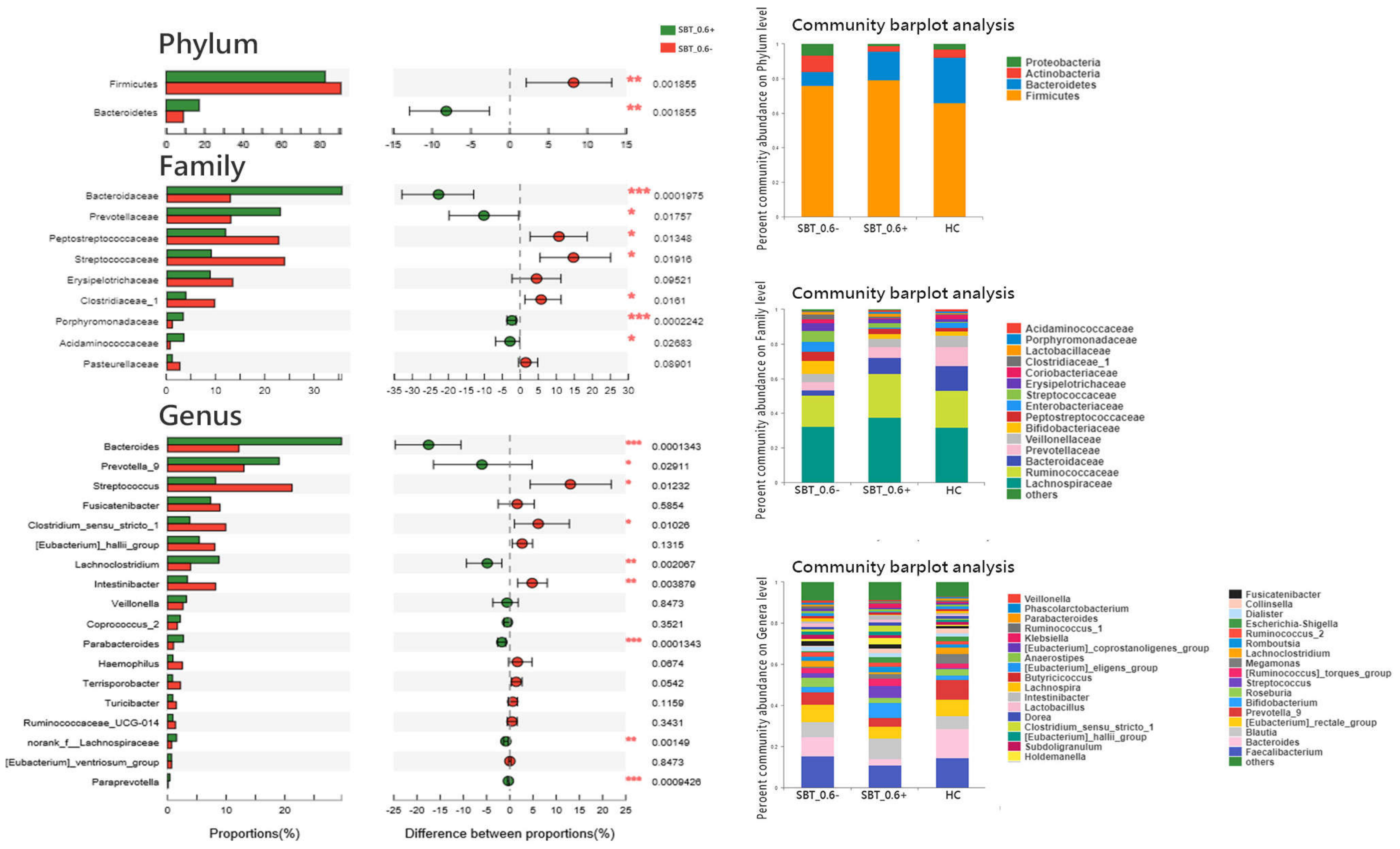

Figure S5. Comparisons of the relative bacterial abundance at phylum, family and genus levels between SBT_0.6+ and SBT_0.6- group, Wilcoxon rank-sum test, *p<0.05, **p<0.01, ***p<0.001. Composition of bacterial taxa on phylum, family and genus level in HC, SBT_0.6+ and SBT_0.6-. Only relative abundances greater than 1% were included. All OTUs with lower abundances were grouped as "other".
HC (n=20), SBT_0.6+ (n=21), SBT_0.6- (n=15)
