# Supplementary material for: Small Bowel Transit and Altered Gut Microbiota in Patients With Liver Cirrhosis
Source: Front Physiol. 2018 May 1;9:470. doi: 10.3389/fphys.2018.00470 (PMC5946013; doi:10.3389/fphys.2018.00470)
Supplement: Supplementary file 12 [file Image_6.PDF]

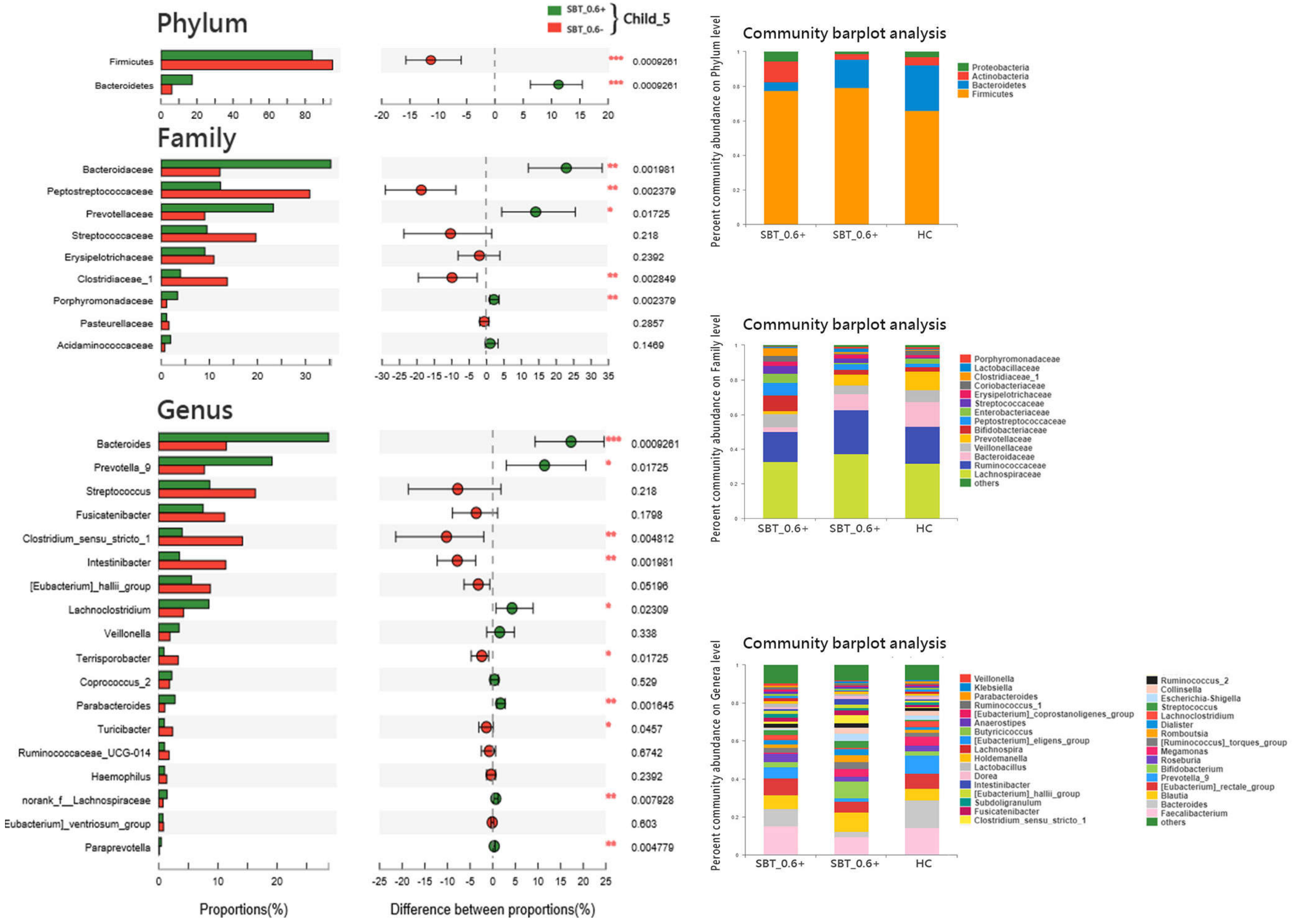

Figure S6. Comparisons of the relative bacterial abundance at phylum, family and genus levels between SBT_0.6+ and SBT_0.6- within Child_5 group, Wilcoxon rank-sum test, *p<0.05,**p<0.01, ***p<0.001. Composition of bacterial taxa on phylum, family and genus level in HC, SBT_0.6+ and SBT_0.6- within Child_5 group. Only relative abundances greater than 1% were included. All OTUs with lower abundances were grouped as "other". HC (n=20), SBT_0.6+ (n=14), SBT_0.6- (n=11)
