# Supplementary material for: Small Bowel Transit and Altered Gut Microbiota in Patients With Liver Cirrhosis
Source: Front Physiol. 2018 May 1;9:470. doi: 10.3389/fphys.2018.00470 (PMC5946013; doi:10.3389/fphys.2018.00470)
Supplement: Supplementary file 13 [file Image_7.PDF]

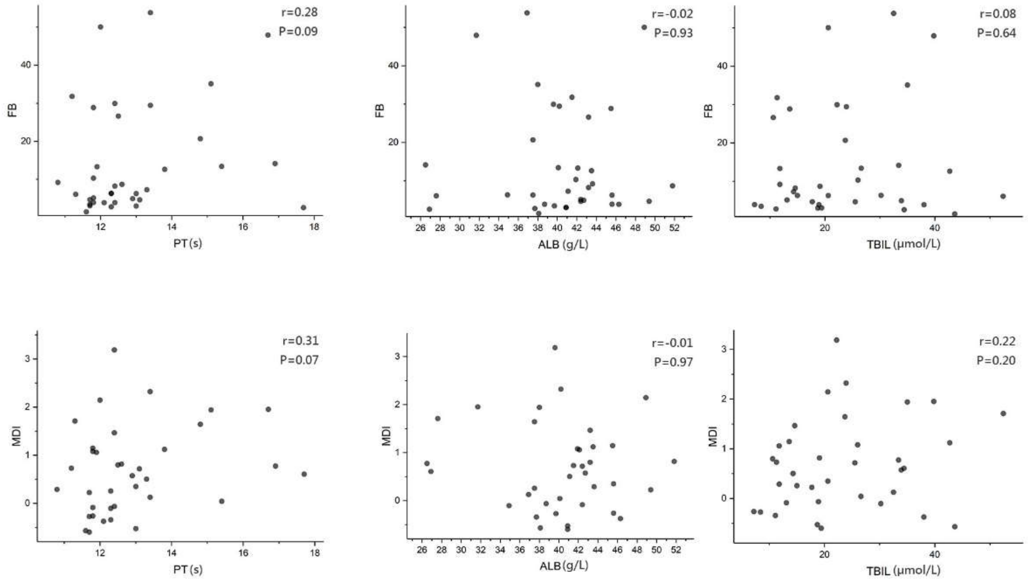

Figure S7. Correlation analysis in LC between (PT, ALB and TBIL) and (F/B ratio and MDI), Spearman's rank test.
LC (n=36).
